# Coupling Effects of Cross-Corticomuscular Association during Object Manipulation Tasks on Different Haptic Sensations

**DOI:** 10.3390/neurosci4030018

**Published:** 2023-08-15

**Authors:** Cristian D. Guerrero-Mendez, Cristian F. Blanco-Diaz, Hamilton Rivera-Flor, Alberto F. De Souza, Sebastian Jaramillo-Isaza, Andres F. Ruiz-Olaya, Teodiano F. Bastos-Filho

**Affiliations:** 1 Postgraduate Program in Electrical Engineering, Federal University of Espirito Santo (UFES), Vitória 29075-910, Brazil; cblanco88@uan.edu.co (C.F.B.-D.); hamriver@gmail.com (H.R.-F.); teodiano.bastos@ufes.br (T.F.B.-F.); 2 Department of Informatics, Federal University of Espírito Santo (UFES), Vitória 29075-910, Brazil; alberto@lcad.inf.ufes.br; 3 School of Medicine and Health Sciences, Universidad del Rosario, Bogotá 111221, Colombia; sebastian.jaramilloi@outlook.com; 4 Faculty of Mechanical, Electronic and Biomedical Engineering, Antonio Nariño University (UAN), Bogotá 110231, Colombia; andresru@uan.edu.co

**Keywords:** corticomuscular connectivity, motor sensory rhythms, mutual information, power-based connectivity, object manipulation, hybrid brain-computer interface

## Abstract

The effects of corticomuscular connectivity during object manipulation tasks with different haptic sensations have not been quantitatively investigated. Connectivity analyses enable the study of cortical effects and muscle responses during movements, revealing communication pathways between the brain and muscles. This study aims to examine the corticomuscular connectivity of three Electroencephalography (EEG) channels and five muscles during object manipulation tasks involving contact surfaces of Sandpaper, Suede, and Silk. The analyses included 12 healthy subjects performing tasks with their right hand. Power-Based Connectivity (PBC) and Mutual Information (MI) measures were utilized to evaluate significant differences in connectivity between contact surfaces, EEG channels, muscles, and frequency bands. The research yielded the following findings: Suede contact surface exhibited higher connectivity; Mu and Gamma frequency bands exerted greater influence; significant connectivity was observed between the three EEG channels (C3, Cz, C4) and the Anterior Deltoid (AD) and Brachioradialis (B) muscles; and connectivity was primarily involved during active movement in the AD muscle compared to the resting state. These findings suggest potential implementation in motor rehabilitation for more complex movements using novel alternative training systems with high effectiveness.

## 1. Introduction

The primary motor cortex plays a crucial role in planning and executing movements by sending signals through the corticospinal pathway to the muscles [1]. Corticomuscular connectivity refers to the statistical dependence between cortical events in the brain and muscle activity [2,3,4]. Electroencephalography (EEG) and Surface Electromyography (sEMG) signals are commonly used to measure corticomuscular connectivity, as they reflect changes in power associated with specific movements, influencing the connections between the brain and muscles [2,5]. However, these measurements depend on the specific task and limb involved [6].

During upper limb movements, neural circuits facilitate the transmission of cortical impulses from the brain to the muscles, establishing functional connections between these systems [2,7]. Precision tasks involving upper limb movements, such as grasping objects, arm segment displacement, and modulation of grasping force based on surface texture and weight, further contribute to the complexity of these neural structures [6]. Additionally, neuronal rhythm randomness and the complexity of tasks performed in Activities of Daily Living (ADLs), like object manipulation, can significantly increase the complexity of signals and pose challenges for interpretation [3,8,9].

Corticomuscular connectivity studies have provided valuable insights into how somatosensory information is transmitted from the central nervous system to the muscles. Such studies have also led to advancements in Brain-Computer Interfaces (BCIs), particularly hybrid BCI (hBCI) systems that combine EEG and sEMG signals to improve accuracy and human-machine interaction [2,10]. Motor rehabilitation focusing on upper limb movements is of particular interest to the scientific community due to their importance in ADLs [5,11]. Object manipulation, a complex task involving various sequential movements such as reaching, grasping, lifting, holding, translation, and replacing, remains understudied regarding the coordination and precision changes between the brain and muscles [12,13].

Previous studies utilizing linear coherence-based methods have reported changes in corticomuscular connectivity during object manipulation tasks with varied object weights [5]. These studies indicate increased connectivity with decreased weight and highlight the prevalence of connectivity in the beta (β, 14 to 30 Hz) and gamma (γ 30 to 50 Hz) frequency bands [14,15], increasing connectivity when performing movements involving sustained motor contractions [1], such as those present in lifting and holding objects. Furthermore, previous study have also reported that in the γ band connectivity is increased during dynamic movements or during force enhancement [16,17,18]. Kim et al. [2] have reported that the association between the brain and muscles is present when active and passive exercises are performed with the upper limbs based on the intention of the movement. In contrast, Gao et al. have quantified connectivity for position maintenance and object manipulation [3], and in other previous studies have used connectivity for movement identification, where accuracy has been improved, as shown in [19,20]. Moreover, synchronization between brain and muscle neurons is primarily observed during the execution of motor tasks [21].

Corticomuscular connectivity can be estimated using linear Power-Based Connectivity (PBC) or entropy-based measures such as Mutual Information (MI) [2,3,22]. These methods provide insights into how the cerebral cortex controls muscle activity and enables investigation of the communication between the primary motor cortex and muscles through corticospinal pathways [1]. The cortical events are transmitted to the periphery, while the motor cortex also receives peripheral information.

However, the effects of corticomuscular connectivity during object manipulation tasks involving different haptic sensations (contact surfaces) remain unclear. Understanding these effects is essential for comprehending the communication processes between the central nervous system and muscles during coordinated and synchronized movements. Furthermore, the findings from this study may contribute to the development of hBCI systems for restoring complex movements considering the variations of the environment based in non-ideal conditions.

In this study, cortical events are characterized using the relative power between rest and task, and computing the Event-Related Desynchronization (ERD). This characterization allows the increased cellular excitability in thalamocortical systems that occurs at low amplitude, highlighting the duration of cortical activity in motor tasks [23,24]. Additionally, muscle signals from sEMG are also characterized to determine the electrical activation of each muscle. Subsequent to signal characterization, corticomuscular connectivity is estimated using PBC and MI methods by evaluating different methodologies varying the frequency bands, the influence of EEG channels and muscles, the variation of connectivity with respect to time, among others. Finally, this study evaluates three different haptic sensations during object manipulation, which vary between Sandpaper, Suede and Silk, to assess the variation of connectivity between smooth and rough surfaces.

## 2. Materials and Methods

### 2.1. EEG-sEMG Dataset

In this study, an open dataset called WAY-EEG-GAL, which contains EEG and sEMG signals, as well as data on grip strength and hand position, was used from 12 healthy subjects (8 females and 4 males, aged 19 to 35) [25]. The EEG signals were recorded from 32 channels using an ActiCap device, at a sampling frequency of 500 Hz, located at Fp1, Fp2, F7, F3, Fz, F4, F8, FC5, FC1, FC2, FC6, T7, C3, Cz, C4, T8, TP9, CP5, CP1, CP2, CP6, TP10, P7, P3, Pz, P4, P8, PO9, O1, Oz, O2, PO10 according to the 10–20 international standard system. On the other hand, the sEMG signals were recorded from five different muscles (Anterior Deltoid (AD), Brachioradialis (B), Flexor Digitorum (FD), Common Extensor Digitorum (CED), and First Dorsal Interosseous (FDI)) at a sampling frequency of 4 kHz.

During the experiment, participants were instructed to rest their right upper limb on a table for 2 s. Then, a LED light provided a visual cue for them to start reaching towards an object with their right hand. They had to grasp the object with their index and thumb fingers, lift it, and hold it steadily within a circle approximately 5 cm above the table for 2 s until the LED light turned off. After that, they had to replace the object and return their upper limb to the starting position. The objects used in the experiment varied randomly in two different conditions: weight and contact surface. The weight variations were between 0.165 kg, 0.330 kg, and 0.660 kg, and to modulate the weight, an electromagnet was used. For the variation of the contact surface (Sandpaper, Suede, and Silk), an external person intervened to change it. The experiment consisted of ten series of approximately 32 trials each, resulting in a total of 328 trials per participant, where the weight of the object, contact surface, or both were changed.

**Figure 1:**
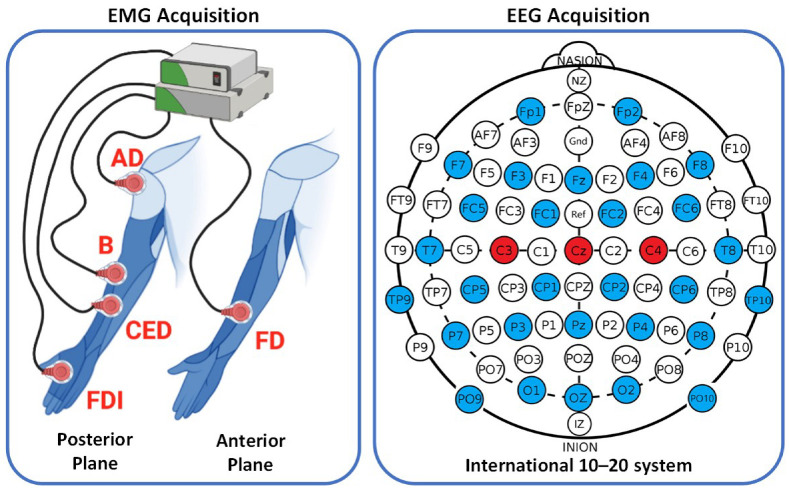
Location of sEMG and EEG Channels (red color) used in this study.

To compute corticomuscular connectivity, this study used three EEG channels (C3, Cz, and C4) and five sEMG channels, as presented in Figure 1. Data from three different surface contact were used when the subject manipulated the object (sandpaper, suede, and silk) where the object weight was kept constant on 0.330 kg. Two contact series were conducted, each consisting of 33 trials, with 11 trials per surface contact, totaling 66 trials per participant. Data were used for each trial until the subject completed the task of replacing the object.

### 2.2. Signal Pre-Processing

To ensure that the EEG and sEMG signals had the same data size, the EEG signals were resampled to match the sEMG sampling frequency of 4 kHz, it has been previously reported [5,26]. Next, the EEG signals were filtered using a Common Average Reference (CAR) filter to eliminate common noise sources from all EEG electrodes, and an eighth-order zero-phase Butterworth filter within the frequency range of 6–50 Hz was applied. The sEMG signals were also filtered using an eighth-order zero-phase Butterworth filter within the frequency range of 20–150 Hz. For both signals, a Notch filter was used to remove line noise at 50 Hz. Only the signals recorded during movement execution were analyzed, as muscle activation during rest is negligible and coherence during resting is generally considered insignificant according to some authors [2,5].

Additionally, in this study, a trial rejection process was employed to identify artifacts in EEG signals using threshold criteria. Specifically, the EEG signals were expected to fall within the range of ±350 μV, as indicated in previous studies [27]. Any trials containing high outliers outside of this range were rejected. If outliers were detected in one EEG channel, the trial was rejected for all EEG and sEMG channels. To ensure an adequate number of trials, in each subject the number of trial rejections should not exceed of 10%; if this range was exceed the subject was rejected. Finally, all participants were included in the study.

### 2.3. Signal Analysis

Cortical events related to spectral power were analyzed for each participant through the analysis of relative power in the frequency domain, and ERD patterns with significance at 0.05. Additionally, the average envelope of the sEMG signal that relates muscle activation during the task was analyzed. This analysis contributes to the understanding of corticomuscular connectivity and extends the findings found in this study. Each method in this analysis was implemented as follows.

#### 2.3.1. Relative Power

The relative power on the three contact surfaces was compared between the baseline (0 s to 2 s, before the LED turned on) and the executed motion (>2 s). For this, the Power Spectral Density (PSD) calculated on the 3 EEG channels was extracted using the Fast Fourier Transform (FFT) with a 1-s Hanning window overlapped at 50% in each frequency band (Mu (μ), Beta (β), Gamma (γ), from 8 to 50 Hz—All). To calculate the relative power between the baseline and the task, Equation (1) was applied, where *A* is the power of the executed movement and *B* the power of the baseline.

(1) RelativePower=(A−B)/B×100

Additionally, a two-sample *t*-test was used to compare the cortical effects related to power between the different contact surfaces. For this, the normality and homogeneity of the data were verified using the Kolmogorov-Smirnov and Levene test. Subsequently, boxplots of each contact surface and the three EEG channels were obtained for statistical comparisons considering a threshold value of p< 0.05.

#### 2.3.2. Significant ERD Patterns

ERD patterns were calculated for each participant in each trial performed for the three contact surfaces. These were calculated on the three EEG channels used for the connectivity analysis. This method was applied to identify the attenuations of short-duration EEG rhythms in the time-frequency domain. Thus, it was applied to the whole movement segment, including the baseline and the executed movement to analyze the distribution in the frequency bands (μ, β, and γ). The ERDs were calculated by implementing a sliding time window of 50 ms, at frequency intervals of 1 Hz using the Morlet Wavelet method in 5 cycles for the time-frequency representations. The ERDs are related to the decrease in relative power between the execution segment and the baseline period (0 to 2 s). For this, Equation (1) was applied. Significant ERD patterns were extracted using the Bootstrap t-percentile algorithm considering a significance level of 0.05. Finally, the significant ERD obtained are presented in time-frequency plots.

#### 2.3.3. Muscle Activation Analysis

The muscle activation of each participant was analyzed in the three contact surfaces when performing the object manipulation task. For this, the signal envelope was extracted using the Root Mean Square (RMS) in the segment of the data where the movement was executed. This method was implemented with a sliding window of 300 ms overlapped at 50%. Additionally, a statistical analysis using boxplot and Analysis of Variance (ANOVA) with Bonferroni *post*–*hoc* test was applied to identify if there were significant differences in muscle activation on each of the contact surfaces for each muscle with a threshold criterion of *p* < 0.05. This method was applied because the data presented a normal distribution, which was verified using the Kolmogorov-Smirnov test.

### 2.4. Corticomusucular Connectivity Methods

#### 2.4.1. Power-Based Connectivity

The Power-Based Connectivity (PBC) was used to estimate the brain and muscles connection during the reach-to-grasp movements. The process was carried out by analyzing the frequency domain of the data using Welch’s method, which is a technique for estimating the Power Spectral Density (PSD). The PSD method consists of dividing the data into overlapping segments and applying a Hamming window to each segment to reduce variability. A modified periodogram was calculated for each segment, and the PSDs of different segments were then averaged to obtain the final estimate. The EEG signals were segmented into 1-s time series with a 50% overlap, and features were extracted in four frequency bands: μ, β, γ, and All band from 8 to 50 Hz, whereas the sEMG features were extracted in the frequency range of 20 to 150 Hz.

In order to estimate the corticomuscular connectivity using PSD features, it is important to adjust the Frequency Resolution (FR) used for each frequency band of the EEG signal to match that of the sEMG signal. This is necessary because the PSD feature vector size for the sEMG signal and each frequency band of the EEG signal must be the same to implement the correlation method. To achieve this, the FR of the sEMG signal was set to 3 Hz, and to estimate the new FR of each EEG frequency band Equation (2) was applied. In this equation, EBL is the individual length of each EEG frequency band with a FR of 1 Hz, and MBL is the length of the frequency band from 20 to 150 Hz with 3 Hz resolution in the sEMG signal. After applying the equation, the frequency resolution was 0.16 Hz for the μ band, 0.37 Hz for the β band, 0.65 Hz for the γ band, and 1.25 Hz for the entire spectrum (All).

(2) $$FR=\frac{EBL-1}{MBL-1}$$

Afterward, the PSD features were normalized using the Min-Max normalization technique, as presented in Equation (3). This ensures that the PSD features remain at the same scale (ranging from 0 to 1) for facilitating the correlation between signal features [19].

(3) $$PSD_{n}=\frac{PSD_{i}-PSD_{min}}{PSD_{max}-PSD_{min}}$$

Finally, the connectivity was estimated by comparing three EEG channels with five sEMG channels, resulting in 15 possible channel combinations. To determine which correlation method used based on the feature data conditions, the normality of the PSD feature data were checked using the non-parametric one-sampled Kolmogorov-Smirnov (KS) test. Then, the Spearman Correlation Coefficient (SCC) was used to estimate the connectivity between the signals. This method was chosen because the feature data for both EEG and sEMG were not normally distributed, and SCC is less sensitive to outliers [28]. Additionally, the *p*-value was calculated with a significance of 0.05 for each correlation using the hypothesis test to determine that the connectivity value is significantly greater than zero, i.e., to evaluate if the connectivity between the signals is significant and not produced by chance.

#### 2.4.2. Mutual Information

Considering the limitations for the interpretation of complex brain-muscle networks by analysis using linear methods, and with the aim of extending and corroborating the findings, this section describes the implementation of Mutual Information (MI) for corticomuscular connectivity estimation [2,3,22]. This method is applied following the descriptions made by [22].

The signals were segmented into adjacent 100 ms windows in the data spanning from baseline to the motion executed when the subject performed the object replacement task. Subsequently, the MI changes between the signals were computed by sliding between the time windows.

The entropy of each signal was calculated using Equation (4) in each time series. For this, first, the optimal number of bins for each signal was determined using Equation (5) based on the Freedman-Diaconis rule. To compute the entropy of continuous data, the data are grouped in a histogram according to the number of bins found, and then the probability that a data value is in each interval is calculated, i.e., the bins values of the grouped data divided by the sum total of all bins values.

(4) $$H\left(X\right)=-\sum_{i=1}^{n}p\left(x_{i}\right)log_{2}p\left(x_{i}\right)$$

(5) $$Bins=\left[\frac{max(x)-min(x)}{3.5\cdot std\left(x\right)\cdot n^{-1/3}}\right]$$

For Equation (4), *p* is the probability of observing the ith value of the data bin of data *x*, *n* is the number of bins. On the other hand, for Equation (5), max(*x*) and min(*x*) are the maximum and minimum values of each signal in each time window, std(*x*) is the standard deviation, and *n* is the number of samples.

Additionally, to measure the information generated by both EEG and sEMG signals, the joint entropy is calculated using Equation (6), where *m* and *n* are the respective number of bins for each signal.

(6) $$H\left(X,Y\right)=-\sum_{j=1}^{m}\sum_{i=1}^{n}p\left(x_{i},y_{j}\right)log_{2}p\left(x_{i},y_{j}\right)$$

Finally, to calculate the mutual information between the signals, Equation (7) is used, which relates the entropy of each signal and the joint entropy between the signals. In this approximation of the computation of mutual information, some delay time in the transmission of the connection was not taken into account, because an analysis of the relationships in the transmission of information between the signals was not addressed, but an analysis of connectivity between the information of the two signals. The computation was performed for each repetition on the three contact surfaces.

(7) MI(X,Y)=H(X)+H(Y)−H(X,Y)

### 2.5. Surface Contact Connectivity Analysis and Statistical Analysis

Four different studies were performed to analyze the connectivity between the signals, based on a previously performed study [5]. The first three studies are based on the results of the PBC method, and the last one is based on the results of MI. The studies are presented as follows.

1. Frequency bands were analyzed by averaging the sEMG channels for each EEG channel in order to identify significant differences between the contact surfaces in each frequency band.
2. Connectivity was assessed using the mean of EEG channels to identify which muscle connection shows significant differences between contact surfaces in each frequency band.
3. Connectivity in the All frequency band was assessed to determine which muscles are significantly more connected to each hemisphere of the motor cortex and on which contact surface.
4. The MI obtained in each muscle was evaluated by averaging the three EEG channels on each contact surface to determine the distribution of information throughout the entire executed movement to find significantly larger distributions between muscles and contact surfaces.

Statistical analysis was performed to determine whether significant differences exist between contact surfaces, EEG channels, and muscles. Specifically, the alternative hypothesis for the first study is that any of the contact surfaces present significant differences in any of the four frequency bands evaluated. For the second study, the alternative hypothesis is that there are significant differences between the contact surfaces for each of the muscles in the frequency bands tested. In the third study, the alternative hypothesis is that there are significant differences between muscles for each EEG channel tested, in addition to significant differences in connectivity between contact surfaces if the same muscle and EEG channel are tested. Finally, for the fourth study, the alternative hypothesis is that some of the muscles present significant differences compared to the other muscles on the three contact surfaces.

To test these hypotheses, ANOVA was implemented with the Bonferroni test as a post-hoc for multiple comparisons using a decision threshold of 0.05. This method was used because the data in all four studies present a high probability of presenting a normal distribution and homogeneous variances, testing this with the Shapiro-Wilk and Levene test. Additionally, the results are presented using violin plots.

## 3. Results

### 3.1. Signal Analysis

#### 3.1.1. Relative Power

**Figure 2:**
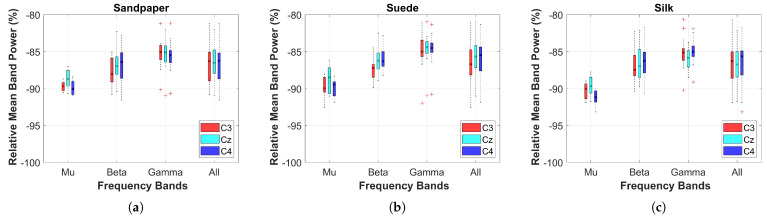
Average relative power of all subjects between rest and task performance on the three contact surfaces. Results are presented for the four frequency bands evaluated in the three EEG channels. The symbol (+) corresponds to outliers in the data. These results are presented for the grand average of all subjects. (**a**) Sandpaper; (**b**) Suede; (**c**) Silk.

The estimated relative power for the EEG channels is depicted in Figure 2. The results show the relative power of the four frequency bands in the three EEG channels for each contact surface. Negative power values indicate a decrease in percentage power, which corresponds to ERD. The μ band exhibits a more pronounced decrease in power compared to the other frequency bands across all contact surfaces (see Figure 2). This effect is consistent across the three contact surfaces. Additionally, channels C3 and C4 demonstrate a greater decrease in power in the μ band compared to channel Cz. However, there are no significant differences in relative power values among frequency bands and contact surfaces (*p* > 0.05). These findings align with previous literature, as the execution of complex tasks activates larger and deeper areas within the cortical motor areas of the brain [5,24]. Furthermore, no significant differences in relative power were observed when comparing each individual channel between the three contact surfaces, except for the comparison between Suede and Silk surfaces in the Cz channel, where a significant difference was found (*p* = 0.048).

#### 3.1.2. Event Related Desynchronization

**Figure 3:**
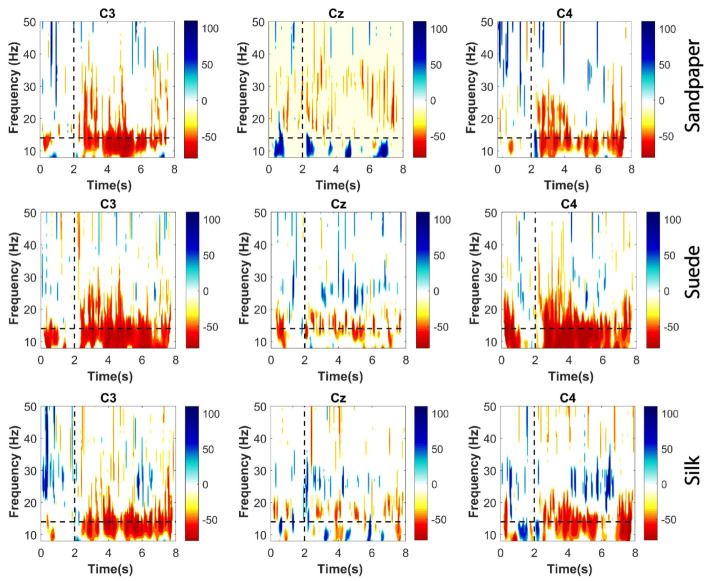
Time-frequency distributions of the significant ERDs on the three contact surfaces and the three EEG channels. The segmentation of the frequency bands is shown in the horizontal dashed lines, and segmentation of task performance (>2 s) and baseline (<2 s) using the vertical dashed line. These results are presented for the grand average of all subjects.

The ERDs computed for each EEG channel on the three contact surfaces are presented in Figure 3. These time-frequency representations encompass the entire EEG spectrum from 8 to 50 Hz and illustrate the grand average of all subjects analyzed. Regarding the significant ERDs derived from the relative power calculations, it can be observed that the μ band exhibits a significant decrease in power throughout the entire movement execution phase following the baseline (LED on). This finding aligns with the results obtained from the relative power analysis, highlighting the pronounced power decrease in the μ band. Additionally, some short time segments demonstrate a decrease in power within the β band. Conversely, certain segments within the β band at high frequencies (20–30 Hz) exhibit power increases, consistent with previous findings reported in the literature [24,29]. Finally, the γ band does not show significant large ERDs, only in some short time segments.

Notably, the Cz channel does not exhibit significant ERD to the same extent as the C3 and C4 channels. This observation can be attributed to the predominant usage of the Cz channel during passive or active movements involving the lower limbs [29]. However, considering the evaluation of a complex movement in this study, which can activate extensive areas of the motor cortex [5,24], both contralateral and ipsilateral cortical activation are involved.

The results presented in this section allow evaluating the power changes in a time-frequency representation, which helps determine at which time instants and frequency bands the cortical effects start to become important.

#### 3.1.3. Muscle Activation

**Figure 4:**
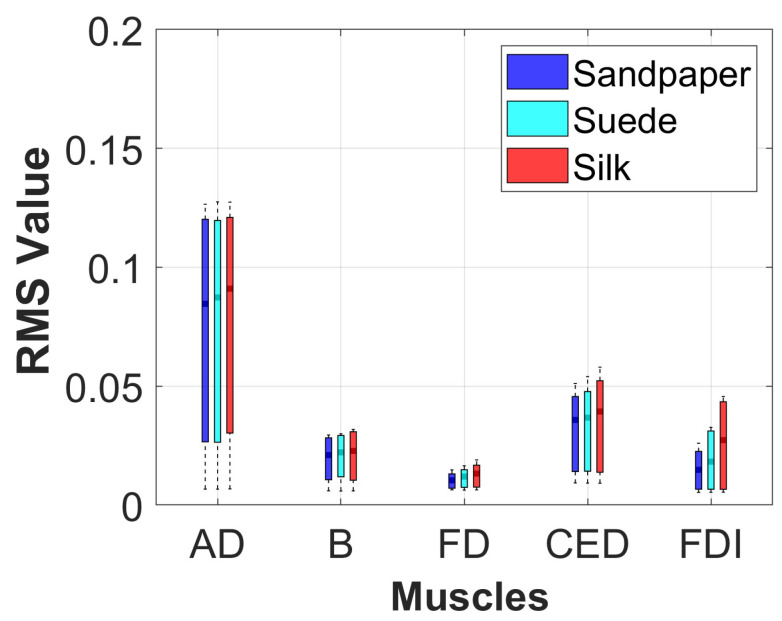
RMS value of each muscle analyzed on the three contact surfaces. These values are presented using the data from the execution of the movement, and using the grand average of all subjects.

The muscle activation results depicted in Figure 4 indicate that the primary muscle involved in the object manipulation task is the AD, exhibiting the highest Root Mean Square (RMS) value. Following AD, the CED muscle demonstrates the second-highest RMS value, followed by muscles B and FDI. The FD muscle exhibits the lowest RMS value. There are no significant differences in muscle activation among the same muscles across each contact surface (*p* > 0.05). However, the boxplots illustrating the results for each surface suggest that the mean RMS value is slightly higher for the Silk surface. Although this difference is not statistically significant compared to the other surfaces, it implies a relatively higher muscle activation level.

### 3.2. Surface Contact Connectivity

**Figure 5:**
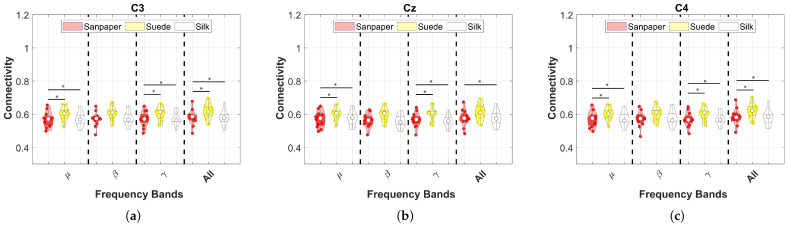
Connectivity results using PBC in the different frequency bands for each EEG channel by averaging the results for the five muscles analyzed. These results are presented for the three contact surfaces. The symbol (*) represents a significant difference with a value of 0.05. The white dot represents the average results of all subjects and the other dots represent the values of each subject. These are the results of the first connectivity analysis. (**a**) C3; (**b**) Cz; (**c**) C4.

According to the studies performed for the analysis of corticomuscular connectivity in contact surface variations presented in Section 2.5, the results for the first study are presented in Figure 5. Basically, the information from the 5 muscles was averaged for each segmented EEG channel in the 3 frequency bands. Each color represents the distributions according to violin plots of the connectivity results, where red represents Sandpaper, yellow Suede, and white Silk.

In the results, it is possible to observe that significant differences are found in the three EEG channels mainly in the μ, γ, and All frequency bands (*p* < 0.05). These differences are mainly centered between the Sandpaper vs Suede and Silk contact surface. Specifically, channels C3 and C4 present the same patterns of significant differences between the aforementioned contacts; however, for Cz, in the All band there are no significant differences between Sandpaper and Suede (see Figure 5). Furthermore, considering the results it is possible to determine that the contact surface with the highest connectivity is Suede for the three EEG channels in each frequency band.

**Figure 6:**
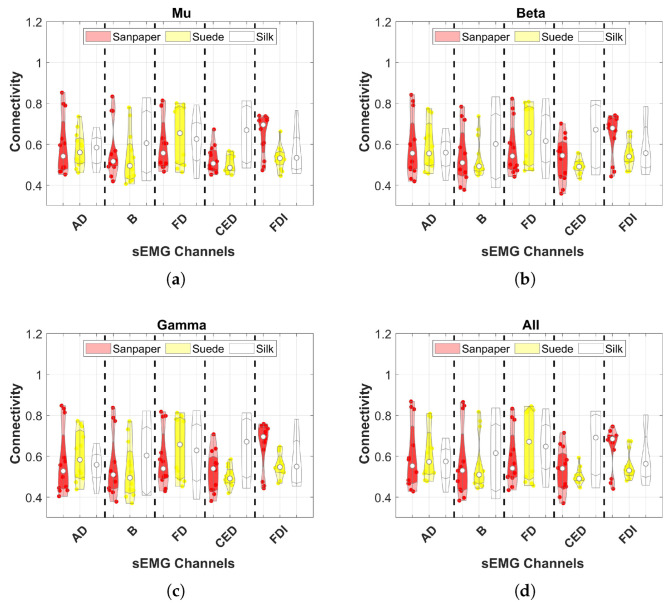
Connectivity results using PBC in the different frequency bands comparing the response in each muscle and contact surface. The three EEG channels were averaged for each muscle, surface and frequency band. This figure shows no significant differences between contact surfaces (*p* > 0.05). The white dot represents the average results of all subjects and the other dots represent the values of each subject. These are the results of the second connectivity analysis. (**a**) μ; (**b**) β; (**c**) γ; (**d**) All.

The second study performed for the connectivity assessment is presented in Figure 6. It averages the EEG channels with respect to each muscle in each frequency band. The results of the contact surfaces are labeled according to the colors red for Sandpaper, yellow for Suede and white for Silk. Each subfigure presents the results for each frequency band evaluating connectivity in the five muscles.

It is important to note that in this study statistical analyzes were performed using violin plots and statistical tests to evaluate differences. However, for each frequency band and muscle, no significant differences were found between the contact surfaces. On the other hand, the behavior of the distribution of connectivity values changes with respect to each muscle, but not with respect to the frequency bands. For example, in the CED muscle the distribution of connectivity values remains compact at a low value close to 0.5 for the Suede surface in the four frequency bands (see Figure 6). In addition, it is determined that the mean of the values is higher for Suede in the FD muscle for all frequency bands.

**Figure 7:**
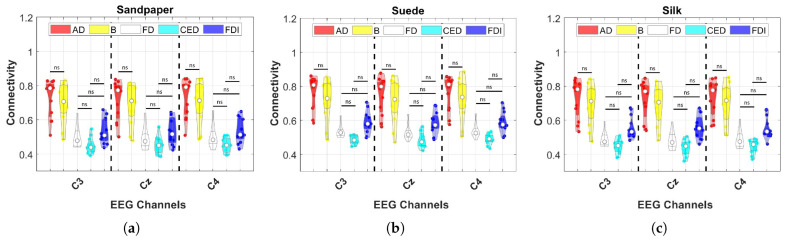
Connectivity results using PBC on the three contact surfaces presented individual connectivity for each EEG channel and muscle combination. These results are presented in the All frequency band. The symbol (ns) represents that there are no significant differences between corticomuscular connectivity (*p* > 0.05). All other comparisons show significant differences. Differences between contact surfaces evaluated using the same EEG channel and muscle were not found (*p* > 0.05). The white dot represents the average results of all subjects and the other dots represent the values of each subject. These are the results of the third connectivity analysis. (**a**) Sandpaper; (**b**) Suede; (**c**) Silk.

Considering that previous studies have evaluated whether there are significant differences between contact surfaces, where differences have been found in certain channels and frequency bands, in the third connectivity evaluation study, the aim is to determine the relationships between muscles and channels. Specifically, contralateral connectivity patterns related to the execution of movements with the right upper limb are looked for. With this, Figure 7 presents the results of the third study where the colors represent each muscle, being red for AD, yellow for B, white for FD, cyan for CED, and blue for FDI. The results are presented for each contact surface, and EEG and sEMG channel in the All frequency band. This band is due to the fact that it takes the whole frequency spectrum analyzed in this study.

According to the statistical results, the muscles that differ significantly in connectivity between other muscles are AD and B, presenting significantly higher connectivity values compared to FD, CED and FDI in the three EEG channels (see Figure 7). Additionally, the muscles where connectivity does not differ significantly are FD, CED and FDI performing multiple comparisons among them (*p* > 0.05). Connectivity also does not differ significantly between AD and B (*p* > 0.05). Finally, as a highlight, comparing connectivity between contact surfaces using the same EEG channel and muscle, there were no significant differences between contact surfaces (*p* > 0.05).

The first three studies were evaluated using PBC, which is a linear method that is used for connectivity estimation, where sometimes the use of linear methods does not allow estimating the nature of complex corticomuscular connections compared to nonlinear methods [22]. Furthermore, with the aim of assessing connectivity throughout task execution in connectivity-time representations, the MI method was implemented in this study to determine connectivity changes between muscles and contact surfaces.

**Figure 8:**
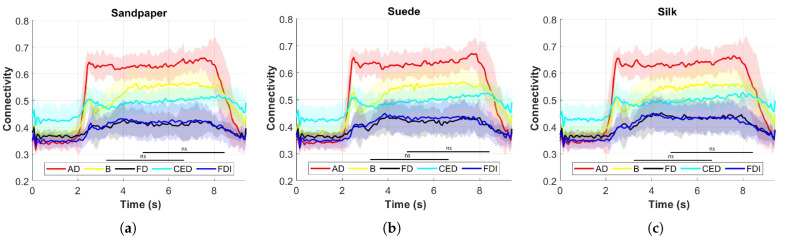
Distribution of MI in each contact surface and muscle analyzed. The mean for each muscle is presented in solid line, and in shaded region, the standard deviation of the calculation between all subjects, according to the color presented. The symbol (ns) represents that there are no significant differences between connectivity (*p* > 0.05). These are the results of the last connectivity analysis. (**a**) Sandpaper; (**b**) Suede; (**c**) Silk.

Accordingly, the MI of the average EEG channels for each muscle on the three contact surfaces during the time of the task from 0 s until the subject performed the object replacing task is presented in Figure 8. Muscles are represented by colors, where red is for AD, yellow for B, black FD, cyan CED and blue FDI. The mean is presented in a solid line, and the standard deviation is in the shaded regions, according to color. It is possible to determine that the AD muscle is the one that presents greater connectivity by MI compared to the other muscles in the three contact surfaces. This difference is significantly greater (*p* < 0.05). The muscles that do not present significant differences between connectivity are B and CED, and FD and FDI (*p* > 0.05). As a highlight, it is impossible to determine that the connectivity estimated using this method presents an increase since the 2 s. This is because at this instant of time the LED is turned on and the volunteers began to perform the experiment. However, at approximately 8 s the subjects began to perform the object replacing task, where it can be observed that the connectivity begins to decrease to approximately the same value previously found before 2 s. These effects are presented in greater proportion for some muscles, being the FD and FDI muscles not so affected in connectivity when executing this type of task.

## 4. Discussion

This study investigates the effects of cross-corticomuscular connectivity during object manipulation tasks by varying the contact surfaces to generate different haptic sensations. The study examines connectivity in three EEG channels and five sEMG channels across various frequency bands by varying the contacts on sandpaper, suede and silk. The main findings of the study are as follows: (a) The PBC values averaged over the sEMG channels exhibit significant differences for each EEG channel between contact surfaces in the μ and γ bands, as well as the All band (8–50 Hz), with suede showing significantly higher values than the other surfaces. (b) When averaging the EEG channels, the PBC values do not display significant differences between contact surfaces, indicating similar means and variances across muscles and frequency bands. (c) EEG channels and muscles showing significantly higher PBC values are C3-AD, C3-B, Cz-AD, Cz-B, C4-AD, C4-B. (d) MI values are significantly higher in the AD muscle, indicating sustained connectivity during task execution compared to the resting state.

Initially, the study analyzes cortical and muscular effects during task execution. Cortical effects are evaluated using relative power analysis to identify significant differences between contact surfaces. Additionally, cortical excitability during tasks is assessed using significant ERD values, which represent short-duration and low-amplitude events. The observed effects align with previous studies on object manipulation tasks involving variations in object weight [5]. The results demonstrate a profound ERD in the C3 and C4 channels, suggesting the complexity of the task based on the relative amplitude and duration of the ERD event, which is particularly prominent after 2 s. The study confirms the task’s complexity, which involves multiple joints and movement synergies, leading to longer and broader neuronal synapses to enhance information processing in peripheral communications [24].

Although the tasks were executed with the right upper limb, an increase in cortical activity in the contralateral and ipsilateral cortices was observed, which was consistent with the findings of previous studies [5,30,31,32]. The aforementioned studies highlight that the complexity of upper-limb tasks may be associated with cognitive tasks or patterns that produce power changes in the contralateral and ipsilateral cortices in the brain, as well as in Cz. However, the connectivity and ERD results of this study demonstrate a greater focus on the C3 channel (see Figure 3 and Figure 5a) for the μ frequency band, which is associated with previous findings in the literature [5,30,31,32].

Different muscle activations were also observed due to the involvement of various joints during task execution. Muscle activation studies revealed that the AD muscle exhibited the highest activation, followed by the CED, B, FDI, and FD muscles. Figure 4 presents the muscle activation results, which vary across muscles due to the distinct muscle synergies required for synchronized coactivation commands.

Previous literature has explored the effects of object manipulation on cortical events in the brain, muscle synergies, and corticomuscular connectivity changes [1,2,3,5,6]. These studies have reported increased connectivity in the β band during object manipulation tasks with varying object weights and holding tasks [3,5]. However, the present study finds relatively similar connectivity values based on PBC across frequency bands (μ, β, γ), although differences between contact surfaces are predominantly observed in the μ and γ bands. Existing literature suggests that the γ band exhibits higher power during sustained and strong contractions [17]. Other studies have shown increased γ band connectivity during dynamic and static forces [18], as well as significant γ band increases in dynamic conditions [3,33]. The findings in the μ band can be attributed to the desynchronization observed during movement, which represents a sustained and deep ERD event during the task.

The variation in contact surfaces during object manipulation tasks significantly influences corticomuscular connectivity, as evidenced by the study’s results. The sensory effects experienced during haptic sensations when manipulating objects contribute to distinct motor behaviors in the upper limbs when contact surfaces are changed. The central nervous system adapts its control and coordination of muscles to meet specific environmental demands. For example, manipulating objects with smooth surfaces requires greater muscle strength and coordination to maintain a secure grip [17,18], potentially resulting in increased connectivity. However, the study reveals a significant difference between Sandpaper and Silk, highlighting greater connectivity between these two surfaces.

Conversely, the study does not find significant increases in connectivity across frequency bands in individual muscles, indicating no specific muscle being more connected to the motor cortex. Moreover, no differences between contact surfaces are observed in the results for each muscle. However, a notable variation is observed in the CED muscle responsible for finger extension, directly involved in object manipulation tasks [25,34]. This muscle plays a role in perceiving receptive stimuli from the environment during haptic sensations. Increased connectivity is reported during haptic sensations elicited by Suede compared to other contact surfaces. Additionally, the three EEG channels exhibit higher connectivity with the AD and B muscles, consistent with previous literature on object manipulation with varying object weights [5].

Mutual Information (MI) analysis is employed to assess how connectivity varies throughout the movement and response in different muscles. Comparing the significant ERD findings with the characterization of cortical responses, it is determined that MI yields coherent results similar to cortical responses, indicating greater connectivity during active movement compared to the resting state [2]. This suggests strong synchronization between cortical events and muscle responses during object manipulation. Moreover, certain muscles exhibit higher MI values, particularly the AD muscle, indicating a strong relationship between cortical signals and muscle responses during object manipulation. These findings support the crucial role of corticomuscular connectivity in coordinating and executing movements during object manipulation.

The reported findings can be further expanded by considering the peripheral responses sent to the brain during different haptic sensations. As contact surfaces change, receptors in the hand and fingers transmit altered peripheral responses to the brain. These responses provide information related to texture, shape, weight, and other aspects relevant to object manipulation, enabling the brain to precisely control objects. Exploring these responses would enhance the understanding of the findings. Methods such as Granger Causality, which has been used to measure peripheral responses in holding and force increase tasks [3], can be employed for such studies.

In summary, this study provides an analysis of corticomuscular connectivity using Power-Based Connectivity and Mutual Information during object manipulation tasks with varying haptic sensations. The study characterizes cortical and muscular responses during movement and conducts in-depth connectivity analyses to assess the influence of cortical events on the synapses between brain and muscle neurons during coordinated movements. The study’s strengths lie in using methods that involve higher energy and power of the sEMG signal across a wide frequency band (20–150 Hz) compared to limited and reduced frequency bands in coherence methods [1,5]. Furthermore, the study employs nonlinear methods to evaluate connectivity responses, supporting and extending the presented results.

The limitations of the study include the lack of exploration of peripheral responses sent to the brain, which would enhance and support the findings. Another limitation of this study corresponds to the analysis performed using only the right upper limb during the execution of complex tasks, and finally, a further limitation can be found in the non-exploration of sex-specific connectivity changes. However, future studies will focus on deepening the corticomuscular activity in tasks using the right and left upper limbs and deepening sex-specific connectivity, which would allow direct comparison with these findings and expand knowledge about the behavior of the neuromuscular system.

## 5. Conclusions

This study investigated corticomuscular connectivity during object manipulation tasks involving varying contact surfaces (Sandpaper, Suede, and Silk) using Power-Based Connectivity and Mutual Information methods with three EEG and five sEMG channels. The results revealed significant differences in the Mu (μ) and Gamma (γ) bands, with the Suede contact surface exhibiting the highest connectivity. Corticomuscular connections were found to be strongest in the C3, Cz, and C4 channels, which were connected to the Anterior Deltoid (AD) and Brachioradialis (B) muscles. Furthermore, the AD muscle demonstrated the highest activation during active object manipulation movements compared to both rest and the connectivity of other muscles. These results have important applications in designing and implementing protocols for motor rehabilitation of lost upper limb movements, particularly for recovering complex movements involved in object manipulation. Additionally, these findings have the potential to significantly improve the performance of BCI systems for upper limb-based motor rehabilitation.

Future studies will focus on investigating bidirectional connectivity to analyze peripheral responses, implementing these findings in BCI systems for motor rehabilitation, and exploring these movements within paradigms such as Motor Imagery.

## Data Availability

The data used in this research were taken from the public database reported in [25].
